# The Timeout in Sports: A Study of Its Effect on Volleyball

**DOI:** 10.3389/fpsyg.2019.02437

**Published:** 2019-10-29

**Authors:** Carmen Fernández-Echeverría, Jara González-Silva, Inma T. Castro, M. Perla Moreno

**Affiliations:** ^1^Faculty of Education, University of Seville, Seville, Spain; ^2^Faculty of Sports Sciences, University of Extremadura, Cáceres, Spain; ^3^Faculty of Sports Sciences, University of Granada, Granada, Spain

**Keywords:** coach management, timeouts, formative stages, volleyball, binary logistic regression

## Abstract

The purpose of this study was to analyse the variables (lost rallies and score difference) that determine the timeout effect (positive or no effect) in volleyball, in balanced and unbalanced sets. 232 timeouts, requested by the coaches of 66 male and female teams participating in the Spanish Championship in the Under-14 and Under-16 categories, were analysed. The variables considered in this study were timeout effects, lost rallies and score differences. To analyse the timeout effect, a binary logistic regression model was applied. The results of this model show that, in balanced sets, the variables that predict the timeout effect are the number of rallies (≤2 lost rallies) and the score difference (2–3 points), whilst in unbalanced sets, and the variable that predicts the timeout effect is the number of lost rallies (3 lost rallies). These results show the importance of bearing these variables in mind when timeouts are managed and requested by coaches, in order to optimise the team’s performance.

## Introduction

In team sports, there are different pauses during competition when coaches can intervene directly with their players. These pauses include timeouts, technical timeouts, substitutions, and the intervals between parts, sets or quarters, depending on the sport modality. Of these pauses, only substitutions and timeouts can be freely managed by the coach (Ferreira et al., 2009). These two tools are considered very important for managing the team during competition.

Timeouts not only permit interrupting the game, but they also permit intervening directly with the players (Díaz and Díaz, 2012) and having a direct impact on the most relevant aspects of the game (Zetou et al., 2008). Therefore, it can be considered one of the most important tools in team sport management that allows coaches to provide direct instructions to their players (Bar-Eli and Tractinsky, 2000; Sampaio et al., 2013).

Due to the importance of the timeouts, the coach must manage them correctly, bearing in mind the available timeouts, requesting them for a specific purpose (Miller, 2005), and transmitting clear, concise and precise information (González, 2007; Estrada and Pérez, 2008). In this sense, the ability to identify in which contexts a timeout must be requested allows the coach to be more effective when managing them, obtaining an improvement in performance of his or her team (Roane et al., 2004; Sampaio et al., 2013; Gomes et al., 2014).

Specifically in volleyball, the regulation allows requesting two timeouts per set to each team, with duration of 30 s. Among the reasons for timeouts requests, we highlight: when the team does not develop the planned tactical and strategic plan; when the team is confused and disorganised; when trying to change the pace of the game and adapt it to one’s expectations (Zhang, 1993; Herrera et al., 1996); to manage fatigue states (Duke and Corlett, 1992); and to influence the psychological/emotional status of the players (Wang et al., 2010). In addition, in volleyball, it is recommended that, before asking for timeout one should think and assess the state or situation of the set, the points that each team has (score) and the number of timeouts requested by one’s own team, and by the rival (Zetou et al., 2008).

Several studies have attempted to indicate the different factors that influence a coach’s request for timeouts. The most noteworthy factors are match status (Sampaio et al., 2013), the score difference (Zetou et al., 2008) and performance in game actions (Duke and Corlett, 1992; Mace et al., 1992; Roane et al., 2004).

More specifically, some authors have tried to determine when timeouts are most frequently requested during a match. In this sense, in volleyball, the study conducted by Zetou et al. (2008) indicates that coaches mainly request timeouts between 9 and 16 points (54%) and between 17 and 21 points (59%), coinciding with the results of García-Tormo et al. (2003), which indicate an equal balance in the frequency of requests for timeouts between the score ranges of 11–15 and 16–20. In other sports such as handball, timeouts are most frequently requested at the end of each part of the match (Sevim and Taborsky, 2004) before starting the final attacks; in basketball, Gómez et al. (2011) indicate that timeouts are more frequently requested during the last 5 min of the match, and, according to Gomes et al. (2014), during the last 10 min of each part.

The score is another of the variables considered when analysing the request for timeouts in sport. In sports such as handball, basketball and volleyball, the largest number of timeouts are requested when the team is behind in the score (Mace et al., 1992; Zetou et al., 2008; Gomes et al., 2014; Gutiérrez-Aguilar et al., 2016).

When coaches request timeouts, they also bear in mind the team’s previous performance. In this sense, several studies conducted in different sports indicate that the largest number of timeouts are requested after carrying out a negative action. More specifically in volleyball, however, the study by García-Tormo et al. (2003) shows that timeouts are mainly requested when the opposite team consecutively obtains between 1 and 3 points, thus trying to prevent the opposite team from obtaining an advantage in the score, in line with the results of Gil et al. (2010), who state that coaches usually request timeouts more frequently after the loss of 3 points in a row. In handball, however, a timeout is requested after receiving two or three goals in balanced matches, and after receiving 4 to 5 goals in less balanced matches (Gomes et al., 2014) or after having carried out a negative action during the last previous possession (Blanco et al., 2012). In basketball, Duke and Corlett (1992) indicate that, after analysing 35 coaches, errors in technical aspects (throws) and tactical aspects (offencive and defencive adjustments) are the most important aspects taken into account when requesting timeouts.

Another very interesting issue related to timeouts is the evaluation of the timeout effect on the players’ subsequent performance (Moreno et al., 2007; Gómez et al., 2011; Prieto et al., 2016). O’Donoghue and Brown, 2009; Sampaio et al., 2013).

In sports such as volleyball, handball or basketball, some authors indicate a positive timeout effect on the team that requested it. These improvements in performance are also indicated in volleyball, where García-Tormo et al. (2003) showed that, in 77.77% of the occasions, it allowed the team requesting the timeout to break the rivals’ streak and recover serve. In basketball, the existence of an improvement in defencive effectiveness (Gomes et al., 2014) and offencive effectiveness (Gómez et al., 2011; Gomes et al., 2014) is observed. Furthermore, Sampaio et al. (2013) and Ortega et al. (2010) hold that teams that request a timeout show an increase in their score and improve their results.

Therefore, timeouts have been investigated in many studies and from different perspectives, but all of them have proved that it is one of the most important tools available to coaches during competition (Zetou et al., 2008; Gómez et al., 2011; Saavedra et al., 2012; Sampaio et al., 2013). However, in volleyball, the reduced number of studies aimed at identifying the contexts in which the timeout is requested, and at knowing its effect on the game, suggests that there is a need to carry out work along this line. Specifically, we believe it is necessary to analyse one of the tools that coaches possess to manage their team during the competition and give them specific indications about the game, the timeouts. On many occasions, depending on whether the score is matched or not, coaches do not know how for many lost rallies they should wait to request timeout or the difference in points at which it is more appropriate to request it so that the use of timeout will have a positive effect. Therefore, the general objective of the study was to analyse the variables (lost rallies and score difference) that determine the timeout effect (positive or no effect) in volleyball, in balanced and unbalanced sets.

The specific objectives were:

– To determine whether the variable lost rallies influences the effect of timeout (positive or no effect) in volleyball, in balanced and unbalanced sets.
– To determine whether the variable score difference influences the effect of timeout (positive or no effect) in volleyball, in balanced, and unbalanced sets.

## Materials and Methods

### Sample

The study sample was comprised of 232 timeouts requested by the coaches from 66 teams (36 female teams and 30 male teams), participating in the Spanish Championship in the Under-14, and Under-16 categories. The choice of this sample was due to the fact that a representative team from each region participates in this Championship, bringing together the best players and coaches of training stages in Spain.

The timeouts correspond to the observation of a match played by each team. This represents the observation of a total of 123 sets.

In this work, both genders and the two categories Under-14 and Under-16, were jointly considered. This was due to the fact that a differentiated analysis by gender and category had been previously performed, finding no significant differences in the effect of timeouts and their management in the different groups. Together with this, the inclusion of the two categories and genders allowed a greater number of timeouts, and therefore, with a larger sample.

The study is exempt from ethical approval because the observation of the game actions does not pose any risk to the participants. It was in accordance with Spanish and international guidelines for scientific research involving humans. The team coaches were contacted in advance, informing them of the confidentiality of the data and guaranteeing their anonymity. The informed and written consent of the participants was obtained for the study.

### Variables

The following variables were considered in our study: (a) timeout effect (adapted from García-Tormo et al., 2003), defined as the consequence on the game of requesting a timeout. Two levels are considered in this variable: positive timeout effect, defined as recovery by the team that requested the timeout of the serve in the first rally in K1 (K1 is known as the attack phase, defined as the situation in which the team that receives the serve sequentially performs the actions of reception, second set and attack), and timeout with no effect, defined as the non-recovery by the team that requested the timeout of the serve in the first rally in K1; (b) lost rallies (adapted from Gil et al., 2010), defined as the number of consecutive moves that occur whilst the opposite team is in possession of the serve until the timeout is requested. Three levels are considered in this variable: ≤2 rallies, 3 rallies, and ≥4 rallies; (c) score difference (adapted from Zetou et al., 2008), defined as the difference in score between the teams when the timeout is requested. Three levels are considered in this variable: ≤1 points, 2–3 points, and ≥4 points.

The score reached at the end of the set by both teams was taken into account when analysing these variables. Thus, grouping the variables used by Drikos and Vagenas (2011), our study differentiates between balanced sets (sets when both teams reach a score of 20 or more points) and unbalanced sets (sets when one of the two teams does not reach a score of 20 points).

### Procedures and Data Gathering

Initially, to guarantee the validity of the instruments, a group of four researchers, experts in volleyball (Graduates in Physical Activity and Sport Sciences, National Level III Volleyball Coaches, with experience as volleyball coaches), developed the observation instrument in agreement with the theoretical framework and based on existing bibliography. The data was initially recorded in Excel and later transferred to the SPSS for analysis. All statistical analyses were per formed using the statistical software package SPSS (2010).

The data were later recorded on video. The matches were recorded using a SONY HDR-XR155 digital camera (M2TS format). This camera was located at one end of the court, at a height of 5 m above floor level and a distance of 7 m behind the baseline, to obtain an optimal line of sight. This position allowed the observation of the two playing fields and the area of the bench, including the coach’s position, to ensure the registration of timeout at the time of its request. In addition, the camera position allowed the observation of the game score.

For the reliability of the observation, after collecting the video footage and prior to the coding process, one experienced observer carried out training processes. In this training process, he observed different training sessions in different situations (balanced and unbalanced sets). The intra-observer Cohen’s Kappa values reached in the observation of all the variables were higher than 0.81 in the fourth training session, which was the minimum value considered to be almost perfect agreement (Landis and Koch, 1977). To guarantee the time reliability of the measurement, the same coding was performed on two occasions, with a time difference of 10 days, obtaining Cohen’s Kappa values higher than 0.81.

### Statistical Analysis

The absence of multicollinearity was verified through the level of tolerance 1-*R*^2^, where *R*^2^ denotes the squared multiple correlation of the random variables lost rallies and score difference, and the Variance Inflation Factor (VIF) 1/(1-*R*^2^). The levels of tolerance were 0.982 and 0.984, and the value of VIFs were 1.018 and 1.016 for balanced sets and unbalanced sets, respectively. As the tolerance values were greater than 0.5, and the values of VIFs were less than or equal to 5 (Kleinbaum et al., 1988), there were no collinearity problems.

A binary logistic regression analysis was performed to predict the behaviour of the dichotomous categorical dependent variable (timeout effect), as a function of the predictive variables (lost rallies and score difference), and to assess the influence of each predictive variable on the response in the form of OR (Odds Ratio). The objective was to obtain a multivariable model, *method used: Enter* (Landau and Everitt, 2004) that would permit knowing the timeout effect on two different situations, balanced and unbalanced sets. The level of statistical significance was set at *p* < 0.05 in all the analyses conducted.

In the binary logistic regression model, the log odds of the outcome is modelled as a linear combination of the predictive variables x_1_, x_2_, …, x_*k*_, with the coefficient vector B = (b_0_, b_1_, b_2_,…, b_*k*_) as follows:

(1) $$Z=\log\left(\frac{p}{1-p}\right)=b_{0}+b_{1}\,x_{1}+b_{2}\,x_{2}+\cdots +b_{k}\,x_{k}$$

where *p* is the probability of the event of interest occurring (in our case, the positive timeout effect). Given the values of the predictive variables, we can estimate the probability of the event of interest as follows:

(2) $$p=\frac{e^{Z}}{1+e^{Z}}$$

where *e* is the Euler number equal to 2.71828, and *Z* is given in Equation (1).

## Results

Table 1, using the test chi-square (χ^2^), shows that, in balanced sets, the number of lost rallies and score difference was significantly associated with the timeout effect, whereas in unbalanced sets, only the lost rallies variable were significantly associated with the timeout effect, due to the fact that the score difference variable did not fulfil the necessary conditions to validly apply the chi-square test.

**TABLE 1:** Association of the independent variables with the dependent variable.

		**χ^2^**	**Sig.**	**Cramer’s V**
Balanced sets	Lost rallies	6.012	0.049	0.219
	Score difference	6.914	0.032	0.235
Unbalanced sets	Lost rallies	6.033	0.049	0.237
	Score difference	0.460	0.794	0.066

### Variables Associated With the Effect of Timeouts in Balanced Sets

The binary logistic regression model in balanced sets presented a significant value for the timeout effect (χ^2^ = 12.872; *odds ratio, exp* (B) = 1.50, *p* < 0.05), with the lost rallies and score difference variables acting as predictors of the positive timeout effect.

The odds ratios indicate that requesting a timeout in balanced sets after waiting for 2 lost rallies or less, instead of 4 lost rallies or more, increases the frequency of the timeout effect being positive by 3.63 times, instead of having no effect. Furthermore, requesting the timeout with a score difference of 2 to 3 points, compared with 4 or more points, increases the frequency of the timeout effect being positive by 2.81 times, instead of having no effect.

### Variables Associated With the Effect of Timeouts in Unbalanced Sets

The binary logistic regression model in unbalanced sets presents a significant value for the timeout effect [χ*^2^* (1) = 6.357; *odds ratio, exp* (B) = 1.05, *p* < 0.05], with the lost rallies variable acting as a predictor of the positive timeout effect. The score difference variable was excluded from the model, as it did not guarantee the necessary conditions to validly apply the chi-squared test.

The odds ratios indicate that requesting a timeout in unbalanced sets after waiting for 3 lost rallies instead of 4 lost rallies or more increases the frequency of the timeout effect which is 4 times more positive, instead of having no effect.

### Probability of the Positive Effect of Timeout, Considering the Variables Lost Rallies, and Score Difference

As shown in Tables 2, 3, the binary logistic regression model estimates the probability (P) of the positive timeout effect in sets of different intensities, using the logistic regression coefficient vector (B) given in Equation (1):

**TABLE 2:** Model of binary logistic regression analysis in balanced sets.

				**95% confidence**	
				**intervals**	
	**Logistic regression**				
**Variables**	**coefficient (B)**	***P***	**OR**	**Lower**	**Upper**
**Lost rallies**					
≤2 rallies	1.292	0.02	3.63	1.182	11.200
3 rallies	0.750	0.30	2.11	0.507	8.839
≥4 rallies^a^					
**Score difference**					
≤1 points	–0.107	0.81	0.89	0.360	1.242
2–3 points	1.036	0.04	2.81	1.047	7.589
≥4 points^a^					
Constant	–0.903	0.128	0.40		

*^*a*^Category of reference for the independent variable.*

**TABLE 3:** Model of binary logistic regression in unbalanced sets.

				**95% confidence**	
				**intervals**	
	**Logistic regression**				
**Variables**	**coefficient (B)**	***P***	**OR**	**Lower**	**Upper**
**Lost rallies**					
≤2 rallies	−0.036	0.93	0.96	0.399	2.328
3 rallies	1.386	0.04	4.00	1.066	15.012
≥4 rallies^a^					
Constant	−0.134	0.715	0.87		

*^*a*^Category of reference for the independent variable.*

(3) $$\begin{array}{l} Z=Ln(P/1-P)=b_{0}+b_{1}Lost\;rallies\_1+b_{2}Lost\;rallies\_2\\ \;\;\;\;\;\;\;\;\;+b_{3}Score\;difference\_1+b_{4}Score\;difference\_2 \end{array}$$

To clarify the previous equation, we present the following example. A team requests a timeout when it has lost two or less consecutive rallies, and with a score difference of 2 to 3 points. Then, from (3), the log odds of the positive timeout effect is equal to:

Z=−0.90+1.292+1.036=1.428

Hence, the probability of the positive timeout effect is equal to e^1.42^ = 4.13.

Using Equation (3), the probability of a positive timeout effect in balanced sets for this value of *Z* is:

p = odds/(1 + odds) = 4.13/5.13 = 0.8050

If the number of lost rallies that occurs until the timeout is requested is less than or equal to 2 and with a score difference of 2 to 3 points, the probability of a positive timeout effect is equal to 80.50% in balanced sets.

For balanced and unbalanced sets, the positive timeout effect probabilities are shown in Tables 4, 5.

**TABLE 4:** Probability of a positive timeout effect in balanced sets.

		**Lost rallies**	
		**≤2 rallies**	**3 rallies**
Score difference	≤1 points	57.02%	71.09%
	2–3 points	80.50%	70.71%

**TABLE 5:** Probability of a positive timeout effect in unbalanced sets.

**Lost rallies**	
**≤2 rallies**	**3 rallies**
71.97%	77.88%

As shown in Tables 4, 5, in balanced sets, we highlight that, when the score difference is 2 or 3 points and the timeout is requested after ≤2 consecutive lost rallies, the probability of a positive timeout effect is 80.50%. In unbalanced sets, when the timeout is requested after 3 consecutive lost rallies, the probability of a positive timeout effect is equal to 77.88%.

The classification table of the model is shown in Table 6. We highlight that, for balanced sets, the positive timeout effect is correctly classified in 90.7% of the cases.

**TABLE 6:** Classification table (balanced sets and unbalanced sets).

**Observed**		**Predicted (Balanced sets)**		
		**Timeouts effect**		
		**Timeout with no effect**	**Positive timeout effect**	**Correct Percentage**
Timeout effect	Timeout with no effect	17	33	34.0
	Positive timeout effect	7	68	90.7
Overall percentage				68.0

		**Predicted (Unbalanced sets)**		
		**Timeouts effect**		
**Observed**		**Timeout with no effect**	**Positive timeout effect**	**Correct Percentage**

Timeout effect	Timeout with no effect	48	4	92.3
	Positive timeout effect	41	14	25.5
Overall percentage				57.9

*The cut value is 0.500.*

## Discussion

The objective of our work was to know the variables (lost rallies and score difference) that determine the timeout effect (positive or no effect) in volleyball, in balanced and unbalanced sets.

Our results show that the score difference acted as a predictor of the timeout effect in balanced sets. More specifically, requesting a timeout in balanced sets, with a score difference of 2 to 3 points instead of 4 or more points, increases by 2.81 the frequency of the timeout effect being positive, instead of having no effect. However, in unbalanced sets, the score difference variable does not act as a predictor of the timeout effect.

Available research, in line with our results, shows that most timeouts are requested with small differences in score (Kozar et al., 1993). The objective of this is, at balanced moments of a match, to interrupt the positive streak of points of the opposite team so that the team requesting the timeout can obtain a positive effect from it, avoiding the accumulation of points of the opposite team without reaction (Mace et al., 1992). More specifically, in volleyball, the study by Zetou et al. (2008) indicated that coaches requested timeouts more frequently when the point difference was two (33%), three (28%) and four (27%). However, this study did not analyse the subsequent effect of the timeouts depending on the score difference. In other team sports such as handball, Gomes et al. (2014) reported that a coach requests more timeouts when there are various negative performances with a matched score (−2 to 1 points) than when the difference in score is greater. Along this same line, in basketball, Gómez et al. (2011) indicated that, when the teams have an equally balanced score (difference between −2 and 3 points), the request for a timeout by the coach produces a better offencive action after this request. However, when the request for timeout is made with large score differences, the offencive performance of the team requesting that timeout does not improve. As e can be observed, the score difference indicated by Gomes et al. (2014) is less than the score indicated by Gómez et al. (2011). These differences may be justified by the scoring system in each sport.

The results of our study show that, in balanced sets, the score difference variable is a decisive factor for the timeout request to have a positive effect. More specifically, a timeout should be requested with a difference of 2 to 3 points. These results suggest that, in balanced sets, coaches must pay attention to this variable when requesting a timeout in order to prevent the opposite team from continuously obtaining points and to prevent the score difference from increasing (Kozar et al., 1993). In contrast, in unbalanced sets, the score difference is not decisive in terms of the timeout having a positive effect after it is requested. In matches with unbalanced sets, these results may be due to the large score differences being clear from the start of the match and to the timeout request not producing considerable changes in the game dynamics (Gómez et al., 2011).

With respect to the variable number of lost rallies, our results indicate that this variable acts as a predictor of the timeout effect in sets of varying intensity. More specifically, if a timeout is requested in balanced sets, after 2 or less lost rallies, the frequency of the timeout effect being positive increases by 3.63, instead of having no effect. Furthermore, if a timeout is requested in unbalanced sets, after 3 lost rallies, the frequency of the timeout effect being positive increases by 4 instead of having no effect.

One of the reasons that lead a coach to request a timeout is not having scored for some time, whereas the opposite team continues to score. The objective of this is to interrupt the rival team’s rhythm (Gomes et al., 2014). Bar-Eli and Tractinsky (2000) pointed out that, during critical moments of the game, for example, due to a consecutive loss of points, players may experience a “state of psychological crisis” that negatively effects their performance, and the coach will have to break the rival’s momentum and recover the performance of his or her players by requesting a timeout during these periods.

More specifically, in line with our results, in volleyball, García-Tormo et al. (2003) and Gil et al. (2010) showed that more timeouts were requested after a loss of 1 to 3 consecutive rallies. Furthermore, García-Tormo et al. (2003) indicated that 77.7% of the timeouts allowed the team requesting the timeout to break the streak of points of the opposite team in the move following this request. These results show that, in formative volleyball, the request for timeouts has a clear influence on the execution of the serve, as a pause in the game may provoke variations in the players’ behaviour. In addition, our work follows the line of other studies carried out in various collective sports such as basketball or handball, which reveal the need to request a timeout after the loss of several consecutive points and after the team’s performing several negative actions (Ortega et al., 2010). Even in spite of not finding volleyball studies that analyse the management of timeouts in situations of equality and inequality in the game, Gomes et al. (2014), in line with our results, analysed performance before requesting the timeout, bearing in mind the situation of the match (matched or not). They found that, during matched moments of the game, timeouts were requested after receiving 2 to 3 consecutive goals, whereas if the match was not matched, the timeout request was made somewhat later, after 4 to 5 consecutive goals. As in our study, this work shows the importance of knowing the team’s situation before requesting the timeout. Thus, when the teams are equally matched, the timeout request must be made earlier on than when there is greater difference between the teams.

The results obtained in our study provide interesting information for managing volleyball competition teams, favouring proper use of one of the tools available to coaches to interrupt the game, timeouts (Zetou et al., 2008). In this sense, the correct decision to request a timeout can be decisive in the development and outcome of the game (Horton et al., 2005). Therefore, we recommend coaches to use this tool strategically, identifying the scenarios that require a timeout according to the score difference and to the number of consecutive lost rallies with the serve in possession of the opposite team, depending on the intensity of the set. This will allow coaches to act more effectively and enhance their teams’ performance (Roane et al., 2004; Gómez et al., 2011).

## Conclusion

The present study shows that, during the direction of the team in competition, in volleyball of training categories, the optimal request for timeout has a positive effect on the team’s game. Specifically, regardless of the intensity of the set, in order to request timeout, the number of rallies lost consecutively (2 or less in balanced sets and 3 in unbalanced sets) must be considered in order to avoid the rival team’s continuous achievement of points and to obtain possession of the serve. However, in balanced sets, it is also necessary to pay attention to the variable score difference (2–3 points), trying to avoid large differences. Hence, the coaches of training stages should consider the possibility of requesting timeouts attending to these variables, in order to manage them optimally.

## Limitations and Future Prospects

In relation to some limitations of the present study, we must state that it was carried out only in training stages, specifically in Under-14, and Under-16 categories. Therefore, it would be interesting to replicate the study in other volleyball game categories (youth and senior or high level) in order to determine whether there are differences in the use and characterisation of timeouts depending on the category. In addition, this study focuses only on the analysis of the use and characterisation of dead timeouts. Therefore, it would be interesting to carry out future studies that combine this information with the analysis of the coach’s verbal behaviour during the course of these timeouts.

## DATA AVAILABILITY STATEMENT

All datasets generated for this study are included in the article/supplementary material.

## Author Contributions

CF-E and MM designed the study. CF-E wrote the original manuscript. All authors critically reviewed and revised the draft, read and approved the final version of the manuscript, and agreed with the order of presentation of the authors.

## Conflict of Interest

The authors declare that the research was conducted in the absence of any commercial or financial relationships that could be construed as a potential conflict of interest.
